# The Neuromagnetic Dynamics of Time Perception

**DOI:** 10.1371/journal.pone.0042618

**Published:** 2012-08-17

**Authors:** Frederick W. Carver, Brita Elvevåg, Mario Altamura, Daniel R. Weinberger, Richard Coppola

**Affiliations:** 1 MEG Core Facility, National Institute of Mental Health, Bethesda, Maryland, United States of America; 2 Clinical Brain Disorders Branch, National Institute of Mental Health, Bethesda, Maryland, United States of America; Duke University, United States of America

## Abstract

Examining real-time cortical dynamics is crucial for understanding time perception. Using magnetoencephalography we studied auditory duration discrimination of short (<.5 s) versus long tones (>.5 s) versus a pitch control. Time-frequency analysis of event-related fields showed widespread beta-band (13–30 Hz) desynchronization during all tone presentations. Synthetic aperture magnetometry indicated automatic primarily sensorimotor responses in short and pitch conditions, with activation specific to timing in bilateral inferior frontal gyrus. In the long condition, a right lateralized network was active, including lateral prefrontal cortices, inferior frontal gyrus, supramarginal gyrus and secondary auditory areas. Activation in this network peaked just after attention to tone duration was no longer necessary, suggesting a role in sustaining representation of the interval. These data expand our understanding of time perception by revealing its complex cortical spatiotemporal signature.

## Introduction

Accurate perception and estimation of time is of fundamental importance to a wide variety of cognitive processes and may underlie numerous motor and cognitive actions. Given the highly complex notion of time, numerous methodologies have been employed to shed light on its central role in human life and our interaction with the ever-changing world. Paul Fraisse eloquently writes “Duration has no existence in and of itself but is the intrinsic characteristic of that which endures” [1](p.2). Other than representing a fascinating metaphysical issue, disturbances in time perception and estimation are associated with a number of neurological and psychiatric illnesses such as schizophrenia [2]–[3] and Parkinson's disease [4]. Determining the neural basis of time perception may provide crucial insight into these disorders. Accordingly, understanding the putative distinction “between a sensorial mechanism for processing duration information and a mechanism that is mediated via a cognitive operation” [5](p. 83) is of interest. Specifically, it has been suggested that there are distinct timing systems in the brain (with different levels of precision) responsible for processing short durations on the order of milliseconds versus long durations counted in seconds or minutes [6], with the former occurring in an automated fashion and the latter requiring more complex cortical attentional/cognitive activation (for reviews [7]–[8]; see also [5], [9]–[11]). The processing of short durations is proposed to rely on primary sensory and motor functions, while long durations may depend on a cognitive timing system that could encompass functions from other cognitive networks.

There is much theoretical discussion concerning the best way to conceptualize the timing problem in cognitive neuroscience [6]. Within the empirical domain there is a sizable literature employing a variety of methods (EEG, ERP, PET, fMRI and MEG) - often in combination- in order to examine brain activation and dynamics [12]–[16]. Not surprisingly vast cortical networks have been implicated in the attention, memory and decision processes that are necessary in timing tasks (for reviews see [17]–[18]). Specifically implicated are the prefrontal cortex, inferior parietal lobule (IPL) and the left supramarginal gyrus (SMG) [19]. A recent study, however, suggests that “the extent of the timing ‘network’ has been significantly over-estimated in the past,” and that with the use of control tasks that are carefully matched for cognitive demands and difficulty, only the inferior frontal gyrus (IFG)/insula, the left SMG and the left putamen are “directly concerned with duration judgments” [20](p. 321). Specifically they - as have others previously - make a strong case for the importance of experimental and control tasks requiring similar cognitive demands, other than the timing component [9], [21]–[25].

Even with careful controls it can be difficult to determine if there are distinct roles for separate brain areas during duration discrimination tasks such as encoding, comparing, and decision making. One promising method for achieving this is to use each unique time course of neural activation during the task as a means of identifying function. Several researchers have employed event-related fMRI for this purpose, In a study by Rao et al. [24] two tones were presented 1200 ms apart (standard interval) followed by a comparison interval that participants were to determine whether it was longer or shorter than the standard. They reported early basal ganglia activation (bilateral caudate and putamen) and right inferior parietal cortex, which they attributed to being uniquely associated with the encoding of time. They suggested that subsequent right dorsolateral prefrontal cortex (DLPFC) activation was involved in comparison of the time intervals. In a further study using two different standard intervals (1200 ms or 1800 ms), Harrington et al. [11] reported findings that suggest two different systems are involved in time perception, namely one supporting interval coding and another for decision making. In this study, the right caudate nucleus, right inferior parietal cortex, and left cerebellum were reported to be involved in interval coding, whereas left middle frontal and parietal cortex were involved in the decision processes. In addition, the left inferior frontal and superior temporal cortex were thought to underlie auditory rehearsal. A more recent study by Harrington and colleagues [26] used longer separation of task components to confirm distinct roles for basal ganglia in encoding and prefrontal cortex in decision making (see also [16], [27]).

Studies that use fMRI or PET to examine timing behavior are limited by the restricted temporal resolution inherent in these methods. Event-related fMRI has resolution on the order of seconds, which requires lengthy separation between components of the task such as encoding and decision making. This may over emphasize processes such as attention and working memory not specifically related to timing. In contrast, EEG/MEG have exquisite temporal resolution, but at least in the past were limited by their spatial acuity. Several researchers have employed EEG/MEG to identify separate cortical processes involved in duration discrimination. Macar and colleagues [28] identified a potential at mesial fronto-central cortex that increased in amplitude with longer target intervals, suggesting a role in a “pulse accumulation” process. A combined EEG/MEG timing study by N'Diaye and colleagues [14] provided evidence of prefrontal activity that differentiates at the offset of a standard duration, implying involvement in the decision process. Paul and colleagues [29] have investigated separate positive and negative electrical potentials that peak at distinct times during and after the second stimulus in a duration discrimination task; furthermore these potentials varied in amplitude depending on task difficulty.

Taken together these event-related fMRI and EEG/MEG studies support the idea that investigating the precise timing of cortical activation during timing tasks can distinguish the role of distinct cortical regions in different phases of the timing process. Modern MEG systems, especially when combined with recent advances in source estimation such as synthetic aperture magnetometry (SAM) used here [30]–[31], have the potential to greatly expand our understanding of spatio-temporal cortical dynamics during time perception. In this MEG study we sought to characterize the real-time dynamics of auditory duration discrimination. Based on work by others [5]–[7], [32]–[33], we expected that the neural networks involved in processing short versus long tones would not be identical (for a review see [7]). Therefore we included separate short and long tone conditions in order to observe both. Each condition consisted of an equal number of two different length tones presented in random order (1200 ms and 600 ms in the long condition; 240 ms and 120 ms in the short). In each case the participant was to press a button after hearing the shorter of the two tones. We also included a pitch detection control condition so as to establish whether there are neural networks involved in auditory time discrimination that are distinct from tone perception and simple response selection. Overall, our goal with this study was to observe the spatio-temporal dynamics of duration discrimination in order to better distinguish the roles of different cortical regions in timing.

## Methods

### Study participants

Twenty healthy right-handed volunteers (mean age 33 (SD 11) years) participated (11 women, 9 men). Informed written consent was obtained from all participants and the study was conducted according to the guidelines approved by the National Institute of Mental Health Institutional Review Board, in accordance with the Helsinki Declaration.

### Behavioral procedures

In each of three conditions participants listened to a series of tones of different durations or pitch. The sine wave tones each had a rise and fall time of 5 ms and were presented binaurally at 75 dB SPL through a pair of low-distortion 5 kHz bandwidth silicone tubes attached to foam ear inserts. In each condition half the tones were targets (shorter tones or lower pitch tones) that required a right thumb button press, and the remaining were non-targets (longer tones or higher pitch tones) that were to be ignored. The non-targets will be referred to here as ‘standard’ tones. There were 80 targets and 80 standards in each condition, with an additional 2 standards at the beginning of each condition that were excluded from further analysis. The order of tones was pseudo-randomized to ensure no more than three tones of either type were presented successively. In the long condition the standard was 1200 ms in duration, and the target was 600 ms. In the short condition, the standard was 240 ms and the target was 120 ms. In the pitch condition the tones were 50 ms in length, the standard frequency was 1.1 kHz, and the target was 1 kHz. All tones were presented at 1 kHz in the short and long conditions. In all conditions the interstimulus intervals (i.e. the intervals between tone offset and tone onset) were randomly jittered between 1000 and 1500 ms. The order of condition presentation was randomized between participants. Participants were given a brief practice before each condition to ensure that they understood the task.

### Data acquisition procedures

MEG signals were recorded continuously in a magnetically shielded room using a helmet-shaped CTF 275-channel whole head magnetometer (CTF Systems Inc., Coquitlam BC, Canada) and sampled at a rate of 600 Hz. For each person, a series of volumetric MRI scans was co-registered with their MEG head coordinate system obtained using fiducial RF coils at the nasion and two preauricular points.

### Data preprocessing

Common mode noise cancellation was applied with 3^rd^ gradient spatial filtering using 30 reference sensors [34]. The data was then high-pass filtered at 0.61 Hz along with DC offset removal. Markers were added at the onset of each type of tone and for finger presses.

### Time-frequency analysis

The raw MEG channel data was analyzed prior to source estimation in order to determine time windows and frequency bands of interest for further investigation. A Time-frequency analysis of the channel data was performed using Stockwell transforms. The Stockwell transform can be thought of as a continuous wavelet transform with a phase correction [35]. Within each condition, a separate analysis was performed for standard and target tones. The time-frequency estimates of signal power were averaged across all channels and trials for each type of tone. The power at each frequency was then normalized by dividing by the average power in a 200 ms pre-stimulus window and taking the logarithm of the result.

### Sliding window SAM analysis

Sliding window SAM analysis was used to determine the spatio-temporal sources of the sensor activity observed in the time-frequency analysis. SAM (synthetic aperture magnetometry), an adaptive beam forming technique, allows the localization of oscillatory power changes, described as event-related synchronization (ERS) or event-related desynchronization (ERD) in specific frequency bands (see [31] for the original formulation of SAM; the method employed here is the vector based approach of [30]). For each predefined voxel location in the brain, SAM creates an optimal spatial filter from the sensor covariance from an ‘active’ time window and a ‘control’ time-window for a specified frequency band. An independent calculation is performed at each voxel location, for the purpose of generating a 3-d source image contrasting the source strengths in the active and control states. Here, independent calculations of event-related changes in source power were performed at a resolution of 7.5 mm cubic voxels throughout the brain volume. The amplitude was determined by computing a pseudo-F ratio between the power in the active and the control states (A/C-1 if A>C, else −C/A+1). Sliding window SAM was accomplished by fixing a 200 ms pre-stimulus control window and performing separate SAM comparisons to 200 ms post-stimulus windows with 50 ms steps. The separate SAM volumes were appended together using Software for Analysis and Visualization of Functional Magnetic Resonance Neuroimages (AFNI) to create a 3d+time dataset [36]. AFNI was also used to align datasets into Talairach space before averaging each condition across participants.

### Group analysis of SAM images

In order to localize regions of peak activation in the 3d+time data, the following procedure was adopted. First, the AFNI program 3dExtrema was used to find the local minima of desynchronization for each time point. Minima were kept for further analysis if they had mean pseudo-F amplitude less than −0.2, which corresponds to 20% greater activation in the control window. In order to ensure significant activation, a whole brain *t*-test across participants was performed at each time point. Local minima were kept for further analysis at a threshold of *p*<0.01. A false discovery rate analysis [37] of all *p*-values from each condition indicated that a *p*<0.01 threshold would produce less than five percent false positives (The 5% threshold was *p* = 0.021 for the long condition, *p* = 0.016 for the short, and *p* = 0.014 for the pitch). As a means of identifying regions of significant activation across time, the remaining minima for each condition were clustered with a connection radius of 11 mm using 3dclust. The locations of peaks in each cluster were determined from AFNI's Talairach atlas.

## Results

### Behavioral data

Responses were considered correct if they came within one second of the end of the target tone. The mean correct percentages were 95, 95, and 94 for the long, short, and pitch conditions, respectively. A single factor ANOVA revealed no significant difference in accuracy between the conditions (*p*>.05). For the long condition, the mean reaction time was 980±65 ms from onset of the shorter tone. The reaction times for the short and pitch conditions were respectively 538±70 ms and 483±76 ms from tone onset. For the tone duration tasks, tone offset is the critical point for decision making; the mean reaction time to tone offset was 380 ms for the long condition and 418 ms for the short condition. A paired t-test between the two conditions showed a significant shorter reaction time to tone offset for the long condition (*p* = .001).

### MEG data

**Figure 1:**
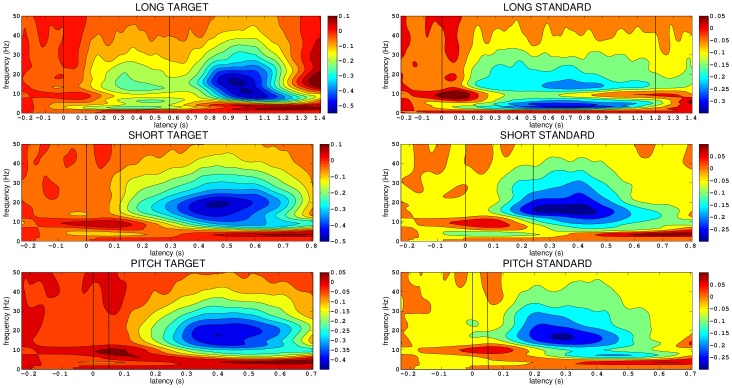
Time-frequency analysis of target and standard tones for each trial type (long, short and pitch).

The time-frequency sensor space analysis of target and standard tones for each trial type (long, short and pitch) is shown in figure 1 (see Methods for details). Red/yellow indicates increased activity relative to prestimulus baseline, and blue/light-blue indicates decreased activity. The scale is the log ratio of post stimulus power to a mean of pre stimulus power at each frequency. The onset and offset of the tones are indicated by vertical lines. Several common features emerge in each plot.

Immediately after tone onset in each graph, there is a brief broad band increase in power, which largely reflects the primary auditory evoked response. For the target tones only there is a large decrease in power (or desynchronization) in the alpha and beta frequency bands (8–30 Hz) near the time of the finger movement. Significantly, during the standard tone, in which there is no movement, a beta-band (13–30 Hz) desynchronization begins just after tone onset in all conditions. For the standard tone, beta desynchronization begins approximately 150 ms after tone onset and continues until 1400 ms, which is 200 ms after the tone ceases at 1200 ms. For the short standard tone, beta desynchronization begins near 150 ms and lasts until 700 ms, well after the tones offset at 240 ms. For the pitch standard tone, beta desynchronization also begins near 150 ms and ends near 550 ms, all of which occurs after tone offset at 50 ms. The following analyses are designed to determine the cortical sources of this activity.

**Figure 2:**
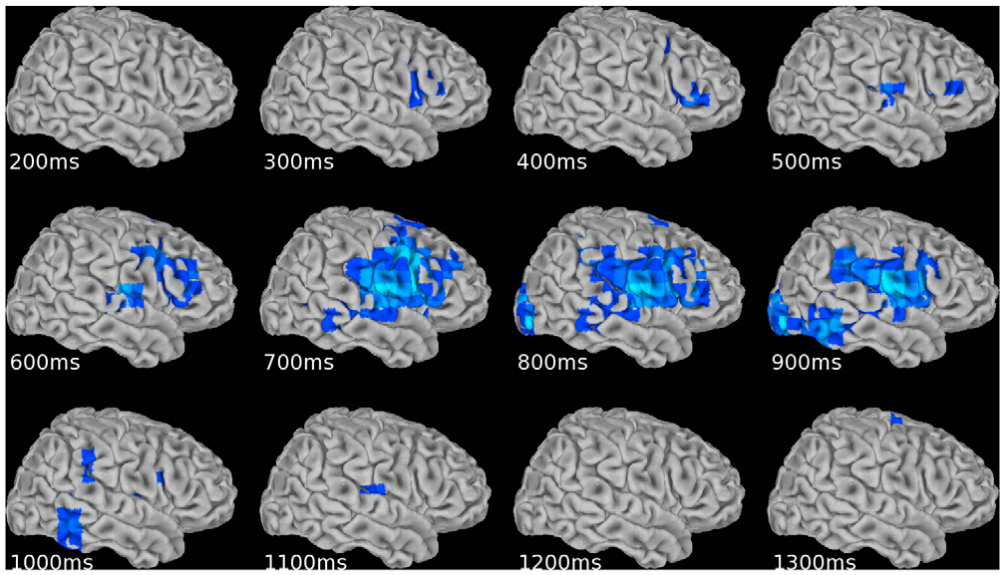
Mean right hemisphere surface maps of beta-band desynchronization (13–30 Hz) during the long condition standard tones.

**Figure 3:**
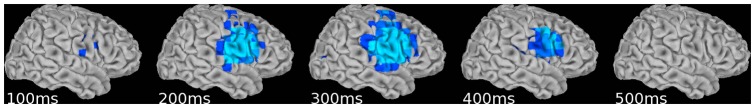
Mean right hemisphere surface maps of beta-band desynchronization (13–30 Hz) during the short condition standard tones.

A sliding window SAM analysis of beta-band activity (13–30 Hz) was conducted for each condition, but only for the standard tones so as to avoid interference from the large amplitude beta desynchronization associated with finger movement. A 200 ms pre-stimulus baseline window was compared to 200 ms post-stimulus onset windows with 50 ms steps. For the long standard tones thirty-nine windows were used – covering an interval from 0 to 2050 ms post-stimulus onset. Twenty windows from 0 to 1100 ms were used for the short standard tones, and sixteen were used in the pitch condition, spanning 0 to 900 ms. Example mean group right hemisphere surface maps of the development of beta-band desynchronization (13–30 Hz) in the long and short conditions are shown in figures 2 and 3 respectively. AFNI SUMA software [38] was used to create the images. The right hemisphere was chosen for display because of greater activity in this hemisphere during the long condition. Activation is thresholded at a mean pseudo-F ratio of −0.2 (see Methods). Lighter blue color indicates greater decrease (or desynchronization) of beta activity relative to prestimulus baseline. The time displayed is the center of a post-stimulus onset sliding window. Not all windows are shown.

Activation appears earlier in the short condition relative to the long (100 ms vs. 300 ms after tone onset). However, the peak of activity in the short condition occurs around 300 ms, after the standard tone has ended at 240 ms. In contrast, in the long condition the most widespread activity occurs between 700 and 900 ms which is during the 1200 ms standard tone. Interestingly this is just after the tone would have ended if it were a target that required a response (600 ms). The pattern of activation in the long condition is temporally more complex and involves a wider array of cortical areas. Beta-band desynchronization appears in right inferior frontal cortex in the window centered at 300 ms. Activity continues in this region up to 800 ms, while spreading to premotor and sensorimotor cortices as well as temporal, parietal and occipital regions. This later activity continues until 1100 ms and is largely gone before the end of the standard tone at 1200 ms.

Figures 2 and 3 present complex spatio-temporal patterns of cortical activity during the standard long and short tones. We sought to systematically categorize the evolution of activity for the standard tones in each condition by performing a cluster analysis procedure described in the methods. The results are presented in tables 1, 2, 3.

**Table 1:** Clusters of beta desynchronization as a function of volume, number of peaks, Brodmann areas, center of mass, and peak latency for the long tones.

Index	Volume (# Voxels/#Peaks)	Brodmann Areas	Center of Mass (RAI Coord.)	Peak Latency (ms)
1	3/5	L 13,43	48.0, 0.0, 13.8	350
2	6/22	L 4,6	45.0, 9.2, 41.0	500
3	6/21	L 3,4,6	31.4, 17.5, 60.9	550
4	5/7	R 6	−19.3, 0.0, 60.9	650
5	18/34	R 6,9,13,22,41,42,43,44,45	−47.4, −2.0, 19.5	700
6	1/1	R 37	−45.0, 60.0, 6.2	700
7	1/3	R 10,47	−37.5, −37.5, −1.2	750
8	1/1	R 24	−7.5, 0.0, 43.8	750
9	1/5	R 18	−37.5, 82.5, −8.8	800
10	5/7	R 22,40	−54.6, 38.6, 29.8	850
11	6/8	L 17,18,19	19.7, 88.1, −0.3	850
12	1/2	R 19	−30.0, 90.0, 6.2	850
13	2/2	R 21,22	−56.2, 45.0, −1.2	900
14	1/2	R 6	−45.0, 15.0, 58.8	1750

**Table 2:** Clusters of beta desynchronization as a function of volume, number of peaks, Brodmann areas, center of mass, and peak latency for the short tones.

Index	Volume (# Voxels/# Peaks)	Brodmann Areas	Center of Mass (RAI Coord.)	Peak Latency (ms)
1	1/6	R 44	−52.5, −7.5, 21.2	350
2	4/9	L 4,6,9,43,44	51.7, 1.7, 20.4	350
3	1/3	R 6	−45.0, 7.5, 21.2	350
4	6/11	L 2,3,4,6	42.3, 18.4, 51.2	350
5	1/1	L 19	22.5, 82.5, −16.2	350
6	1/1	R 18	−30.0, 90.0, −1.2	350
7	1/2	L 17	7.5, 90.0, −1.2	350
8	1/1	R 19	−37.5, 75.0, 6.2	400

**Table 3:** Clusters of beta desynchronization as a function of volume, number of peaks, Brodmann areas, center of mass, and peak latency for pitch.

Index	Volume (# Voxels/# Peaks)	Brodmann Areas	Center of Mass (RAI Coord.)	Peak Latency (ms)
1	2/2	R 6	−56.2, 0.0, 13.8	250
2	2/3	R 6	−32.5, 7.5, 53.8	250
3	1/5	L 6	30.0, 15.0, 58.8	250
4	2/3	R 6,9	−45.0, −2.5, 28.8	300
5	6/6	L 4,6,9,43	48.8, 5.0, 26.2	300
6	4/5	R 40,41,43	−52.5, 19.5, 18.2	300
7	1/1	L 40	37.5, 37.5, 43.8	300
8	2/3	L 3,4	37.5, 27.5, 56.2	350

The local minima of beta desynchronization were calculated separately for each time point. The minima for all time points were then grouped together and clustered to find regions of peak activity. Tables 1, 2, 3 display the Brodmann areas (BA) and number of voxels in each cluster. Also shown are the coordinates for the voxel at the center of mass of each cluster, along with the latency of peak desynchronization for this voxel. A total of fourteen separate clusters appear in the long condition: eight occur in the short and eight in the pitch.

In the pitch condition (table 3), all local minima occur after offset of the standard tone. The earliest activity begins in the 150 ms window and peaks at 250 ms in left and right precentral gyrus and premotor cortex (BA 6). Activity peaks at 300 ms in four clusters encompassing bilateral IPL and pre- and postcentral gyrus, as well as right transverse temporal gyrus (TTG) and right middle frontal gyrus (MFG). A single cluster peaks at 350 ms in left primary motor cortex.

In the short condition (panel B) seven out of eight clusters peak at 350 ms. Peaks at this latency appear in left pre- and postcentral gyrus, bilateral premotor cortex, and bilateral Brodmann area 44 (inferior frontal gyrus (IFG)). Peaks also occur in occipital areas (BA 17, 18 & 19). One additional cluster peaks at 400 ms in occipital area BA 19. Unlike the pitch condition, no clusters appear in the IPL.

The long condition results (panel A) are temporally and spatially more complex than the previous two conditions. One overall difference is a preponderance of activity on the right side; ten out of fourteen clusters on the right, as opposed to four out of eight in both the short and pitch conditions. Figure 2 shows a broad region of right hemispheric activity extending from temporal to prefrontal cortex for the long condition, with the peak between 700 and 800 ms. In the cluster analysis, ten out of fourteen clusters peak between 600 and 900 ms. Of the ten clusters, three appear in occipital areas (left BA 17, 18 & 19 and right BA 18 & 19). All seven remaining clusters with peaks between 600 and 900 ms are on the right side. Of these, the largest (#5 in panel A) contains eighteen voxels and spans latencies from 0.3 to 1.3 seconds (recall that the long tone lasts for 1.2 s). The cluster covers a broad area including voxels in IFG, insula, MFG, pre- and postcentral gyrus, TTG, and STG (BA 6, 9, 13, 22, 41, 42, 43, 44 & 45). Of these areas, activity in BA 13, 22, 42 & 45 are unique to the long condition. Of the remaining six right hemispheric clusters, five also contain areas unique to this condition. These include temporal Brodmann areas 21, 22 and 37; frontal regions BA 10 and 47; and cingulate (BA 24). A cluster spans the temporoparietal junction as well (#10), including superior temporal gyrus (STG), SMG, and IPL (BA 22 & 40). Three of the clusters peaking outside the 700 to 900 ms window originate on the left side, in insula and motor cortex, and peak between 350 and 550 ms. The remaining cluster peaks at 1750 ms in right premotor cortex.

With reference to the left and right cortical surface movies (Movie S1 and Movie S2) that show the time course of beta-band desynchronization (13–30 Hz) during the standard long duration tones, we note that the right hemisphere slides are identical to the fixed images displayed in figure 2. Latencies are the center of a 200 ms sliding window over which beta power is compared to a fixed 200 ms prestimulus window.

**Figure 4:**
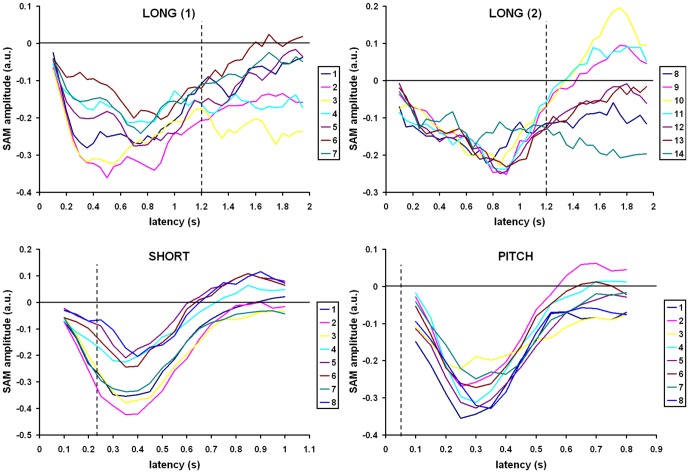
Time series of clusters of cortical activation for the standard tones from each condition.

Figure 4 displays the sliding window time series for the center of mass of each cluster in all conditions. The color coded numbers in the legend refer to the cluster indices from tables 1, 2, 3. Latency is the center of the 200 ms sliding window. The long condition is divided into two graphs for better visibility (long(1) & long(2)). The intent is to provide a more detailed depiction of the temporal evolution of activity in each cluster, more so than the simple peak latencies for the center of mass voxels shown in tables 1, 2, 3. Beta-band desynchronization is indicated by negative values on the graphs, and thus larger negative values imply greater activation. Dashed vertical lines show the offset of the standard tone in each condition.

In the pitch condition beta-band desynchronization increases quickly after tone onset in all clusters, with peak latencies between 250 and 350 ms. Activity returns to near baseline around 600 ms. All clusters are qualitatively similar to each other. This is also the case for the short condition; all cluster time series follow a pattern comparable to the pitch condition with the main difference being an increase in the peak latencies to between 350 and 400 ms and a later rebound time around 700 ms. Also there is a tendency for more clusters to overshoot the baseline and show beta-band synchronization after 700 ms.

As is apparent in tables 1, 2, 3, the time series from the long condition are considerably more complex than the short and pitch conditions. Several of the clusters desynchronize quickly after tone onset with a peak near 300 ms. Some of these then have a distinct second peak near 800 ms, just after the shorter tone would have ended. A second group of clusters desynchronize more slowly and only have a single peak near 800 ms. All but a few clusters rebound to near baseline after tone offset at 1200 ms. Three clusters overshoot baseline by exhibiting greater beta-band synchronization than pre-stimulus, with the most dramatic example in left occipital cortex. As noted earlier, three of the early peaking clusters are on the left side, including primary sensorimotor cortex. All but one of the late peaking clusters are on the right.

## Discussion

Our aim was to employ the enhanced temporal resolution of MEG to elucidate the respective roles of different cortical areas in time perception. Twenty participants each completed three separate conditions. In the long condition participants heard an equal number of 1200 ms and 600 ms tones. They were to respond only to the short tone. Similarly, in the short condition participants heard an equal number of 240 ms and 120 ms tones, the later of which required a button press. In the pitch condition participants heard tones with two distinct pitches, each with a 50 ms duration. They responded to the lower pitch tone. As a first step in analyzing the unaveraged event-related data, we used time-frequency analysis in sensor-space to determine time windows and frequency bands of interest for subsequent source analysis with SAM. Strong event-related beta-band desynchronization was common to all conditions and tone types. Beta-band desynchronization has been associated with cognitive activity in frontal and parietal cortices [39]–[40]. Because it occurs during the tone in all conditions we hypothesized that it is related to cognitive and attentional aspects of the task, including representing and comparing the tones and making the decision to respond. Beta desynchronization has also been associated with motor-related cortical activity. Indeed, a large amplitude beta desynchronization occurs around the time of the button press to the ‘target’ tones in each condition (see figure 1). Because of potential interference from this activity we chose to focus on only the ‘standard’ tones not requiring a button press in each condition.

Sliding window SAM analysis of beta-band desynchronization revealed a distinct spatio-temporal pattern for each condition. A comparison of the short and pitch conditions shows disparate areas of activation (tables 2 and 3). Both conditions are hypothesized to be relatively automatic sensorimotor processes [5]. This is supported by the relative lack of prefrontal activation normally associated with cognition, with the exception of activation in left BA 9 in the short condition as part of a larger cluster extending from motor cortex to IFG, and right BA 9 which clustered with BA 6 in the pitch. IFG activation in the short condition (here bilateral BA 44) is consistent with previous work showing this region involved in timing (e.g. [20]; see also a voxel-wise meta-analysis [41]). The lack of activity in IFG in the pitch condition supports the specificity of the area to timing as opposed to general sensorimotor components of the task.

Occipital activity also occurs in the short but not the pitch condition. In fact, four out of eight clusters appear in bilateral occipital areas. Though occipital activation has been cited in other timing studies (see [10] for a review), especially those employing visual stimuli [42], the function of this region in time perception is unclear. There is no visual component to the current task, although it is possible that some sort of visual imagery is used. A recent study [43] reported occipital activity in the auditory digit span task where a series of digits is heard and repeated. The authors suggest that visualization of the series of digits may be the cause of the occipital activation. However, here it is less obvious what kind of visual strategy might be employed.

Parietal areas arise in the pitch condition but not the short. Bilateral activation in the pitch condition includes a right side cluster centered on the temporoparietal junction encompassing SMG and primary auditory cortex. Activation in this cluster has been observed in fMRI studies of pitch discrimination [44]–[45]. Activation in premotor and motor cortex is apparent in all three conditions, likely reflecting motor preparation and subsequent suppression of the finger movement, given that we performed source analysis on the standard tones, which did not require a button press. In addition, premotor activation may reflect general attentional demands of the tasks [24].

As would be expected, the overall pattern of activation in the long condition is closer to that of the short condition than to the pitch, including clusters in occipital regions. However, there are a greater number of clusters (14 vs. 8), accompanied by activation in several regions unique to the long condition. These include bilateral insula, right temporal areas (BA 21, 22 & 37), right IPL, right anterior cingulate, and right lateral prefrontal and inferior frontal areas (BA 10, 45 & 47). Beside bilateral insula, all the unique regions are on the right, likely reflecting the recruitment of a right side timing network seen in many studies [32]–[33], [46]–[47]. In addition, the unique regions encompass areas known to be involved in cognitive control, working memory, attention, and higher level auditory processing, supporting the involvement of a cognitive system subserving perception of long tones. It is difficult to determine from the current data whether such a cognitive system is an adjunct to an automatic system for perception of short tones, or is instead a distinct system as suggested by some authors [10]; however, it is of interest to note that bilateral IFG activation occurs in the short condition but is limited to the right side in the long condition. Of note, a recent voxel-wise meta-analysis of 41 neuroimaging studies of timing concluded that sub-second tasks in general do tend to recruit more sub-cortical structures than tasks that are supra-second [41]. Moreover, they identified only two regions, namely the right IFG and bilateral SMA, that showed activation at all the interval ranges studied across all the various tasks. Thus, the authors suggest that these regions represent “part of a core network mediating timing across the brain” [41](p.1738).

While the pitch and short conditions produce distinct spatial activation patterns, the time courses of activity in each area are quite similar (figure 4), thus not allowing a clear distinction of their function based on temporal differences. For all clusters of activation, desynchronization begins at tone onset and increases to a single peak after offset of the stimulus before returning to baseline. Given the relatively smooth time courses of beta desynchronization in both figure 4 and the sensor level time-frequency analysis in figure 1, it is doubtful that decreasing the window width and step size of the sliding window SAM analysis (200 ms & 50 ms, respectively) would add temporal information. Rather, the brief stimuli and relatively automatic decision likely leads to a short time course of beta desynchronization.

For the long condition attention must be maintained during each tone for at least 600 ms in order to determine its length. The relatively complex nature of this task is reflected in the varied time courses and distributed peak latencies of beta desynchronization, which suggests distinct roles for the clusters of activation. The earliest peaking clusters occur on the left side in premotor and sensorimotor areas as well as insula (BA 3, 4, 6, 13 & 43). Since a right finger movement is required and the peaks all occur before a possible offset of a short tone (600 ms), these clusters likely reflect attention and motor preparation. Of special interest are two right side clusters (clusters 5 & 7 in table 1) that display a double peak in the time series in figure 4. The first is the largest cluster found in the study, with 18 voxels spanning broad networks including auditory, attentional, and cognitive areas (BA 6, 9, 13, 22, 41, 42, 43, 44 & 45). The second is a smaller cluster in right dorsolateral and ventrolateral prefrontal cortex (BA 10 & 47). The temporal peaks in each cluster occur early after tone onset (300–350 ms) and just after the tone would have ceased if it were a target (700–750 ms). The dual peaks suggest orientation of cognitive and attentional resources to two crucial times; the onset and the potential cessation point of the stimulus when a decision about a button press is required (see [17] for a discussion of orienting attention in time). Two other clusters of interest contain voxels in secondary auditory regions: one in right superior and lateral temporal regions (BA 21 & 22), and the other at the temporoparietal junction, including right SMG (BA 22 & 40). Beta desynchronization in these areas becomes greater in a linear fashion after tone onset until a peak between 850 and 900 ms. These regions are potentially involved in an active store of auditory information during the tone that is no longer needed after the tone is determined not to be a target.

However, given the distributed manner in which the brain operates, parsing of functions may not be especially fruitful. Indeed, recent ideas concerning time representation favor a network model in which the “brain represents time in a distributed manner and tells the time by detecting the coincidental activation of different neural populations” [48](p. 755). The thalamo-cortico-striatal circuits are considered pivotal; especially the basal ganglia, supplementary motor cortex, prefrontal cortex and posterior parietal cortex [11], [19], [24]–[25]. Such a network position reflects a substantial shift in theory (from for example [49]–[50]), but recognizes that this circuitry is not limited to temporal processing since these brain regions underlie many diverse cognitive processes (e.g. working memory), and switching function depends upon the requirements of the task at hand.

Furthermore, Lewis and Miall [47] suggest “that the prefrontal timekeeper function does not rely upon working memory per se, but instead simply draws upon the same neural processes as working memory” (p. 405). Thus, they argue that the very same dorsolateral prefrontal cells are used in both time measurement and working memory because (i) DLPFC is integral to both timing and working memory functions; (ii) both these functions are modulated by dopamine; (iii) during both these functions these neurons modulate their activity in a temporally predictive way; and (iv) this modulation of activation is regulated by dopamine. Such an idea of overlapping prefrontal function in both working memory and timing is compatible with popular notions of prefrontal cortex being a multipurpose processor that is recruited in a wide variety of functions [51]. Conceived as such, the extensive temporal and spatial network observed in our study is not surprising.

It is noteworthy that our time-course analyses revealed right-sided prefrontal activity which for the most part occurred only after the critical 600 ms time-point had passed. It is possible to speculate that this reflects a process related more to the updating of response plans in working memory or to the reorienting of temporal attention, rather than timing *per se*: After approximately 700–900 ms (see Figure 2) the participant may ‘realize’ that the stimulus has not yet stopped and so it must be a 1200 ms standard rather than a 600 ms target. Therefore, at this point the participant can update their response plan (‘no-go’ rather than ‘go’) and, crucially, stop actively timing. In line with this possibility, several studies have already reported that right prefrontal cortex is activated when participants update their response plan as a function of whether a stimulus is, or is not, presented at a critical moment in time [52]–[55]. Indeed, despite the simplicity of timing tasks, the resulting maps of functional connectivity and their implication for cognition are likely to be enormously complex. However, as is evident from these preliminary findings, MEG holds promise of being a critical tool in the elucidation of how this neurocognitive network operates in real-time.

## Supporting Information

Movie S1 **Left cortical surface movie of beta-band desynchronization (13–30 Hz) during standard long duration tones.**
(MPG)

Movie S2 **Right cortical surface movie of beta-band desynchronization (13–30 Hz) during standard long duration tones.**
(MPG)
